# Anopheles ecology, genetics and malaria transmission in northern Cambodia

**DOI:** 10.1038/s41598-021-85628-1

**Published:** 2021-03-19

**Authors:** Amélie Vantaux, Michelle M. Riehle, Eakpor Piv, Elise J. Farley, Sophy Chy, Saorin Kim, Anneli G. Corbett, Rachel L. Fehrman, Anais Pepey, Karin Eiglmeier, Dysoley Lek, Sovannaroth Siv, Ivo Mueller, Kenneth D. Vernick, Benoit Witkowski

**Affiliations:** 1 Malaria Molecular Epidemiology Unit, Institut Pasteur du Cambodge, Phnom Penh, Cambodia; 2 Department of Microbiology and Immunology, Medical College of Wisconsin, Milwaukee, WI USA; 3 Unit of Insect Vector Genetics and Genomics, Department of Parasites and Insect Vectors, Institut Pasteur, Paris, France; 4 CNRS Unit of Evolutionary Genomics, Modeling, and Health (UMR2000), Institut Pasteur, Paris, France; 5 National Center for Parasitology, Entomology and Malaria Control Program, Phnom Penh, Cambodia; 6 School of Public Health, National Institute of Public Health, Phnom Penh, Cambodia; 7 Malaria: Parasites and Hosts Unit, Institut Pasteur, Paris, France

**Keywords:** Ecology, Behavioural ecology, Molecular ecology

## Abstract

In the Greater Mekong Subregion, malaria cases have significantly decreased but little is known about the vectors or mechanisms responsible for residual malaria transmission. We analysed a total of 3920 *Anopheles* mosquitoes collected during the rainy and dry seasons from four ecological settings in Cambodia (villages, forested areas near villages, rubber tree plantations and forest sites). Using odor-baited traps, 81% of the total samples across all sites were collected in cow baited traps, although 67% of the samples attracted by human baited traps were collected in forest sites. Overall, 20% of collected *Anopheles* were active during the day, with increased day biting during the dry season. 3131 samples were identified morphologically as 14 different species, and a subset was also identified by DNA amplicon sequencing allowing determination of 29 *Anopheles* species. The investigation of well characterized insecticide mutations (*ace-1, kdr,* and *rdl* genes) indicated that individuals carried mutations associated with response to all the different classes of insecticides. There also appeared to be a non-random association between mosquito species and insecticide resistance with *Anopheles peditaeniatus* exhibiting nearly fixed mutations. Molecular screening for *Plasmodium sp*. presence indicated that 3.6% of collected *Anopheles* were positive, most for *P. vivax* followed by *P. falciparum*. These results highlight some of the key mechanisms driving residual human malaria transmission in Cambodia, and illustrate the importance of diverse collection methods, sites and seasons to avoid bias and better characterize *Anopheles* mosquito ecology in Southeast Asia.

## Introduction

Malaria remains a major public health challenge with an estimated 229 million cases recorded in 2019^1^. However, while considerable progress has been made in the last decade in decreasing malaria burden, the emergence and spread of parasite drug and mosquito insecticide resistance threaten this achievement^2–6^. To counteract the problem of antimalarial drug resistance, the six countries of the Greater Mekong Subregion aim at eliminating malaria by 2030^7^. Since the launching of the Mekong Malaria Elimination in 2012, a six fold reduction in malaria cases has occurred with Cambodia accounting for 58% of the 239,000 malaria cases reported in 2019^1^.

Malaria control is based on two pillars of rapid diagnostic tests and artemisinin-based combination therapies, and indoor vector control by using insecticide-treated mosquito nets (ITNs) and indoor residual spraying (IRS). Consequently, residual malaria transmission, defined as the forms of transmission that can persist after achieving full universal coverage with effective ITNs and IRS^8^, can be maintained due to mosquito behavioral variation for example, malaria vectors biting mainly outdoors or when people are active. Thus, even an improved coverage and efficacy of IRS and ITNs will not achieve malaria elimination across most of the tropics due to a non-negligible proportion of vectors avoiding fatal contact with these interventions^8^. Residual transmission also maintains the possibility to cause a resurgence of malaria when the vector control program is weakened or withdrawn.

Five classes of insecticides are currently recommended for vector control measures: carbamates, organochlorines, organophosphates, neonicotinoids and pyrethroids, with only the latter being approved for use in ITNs^9^. Following reports of insecticide resistance in malaria vectors, routine resistance monitoring has been set up in several countries. Two types of resistances can be found in vectors: behavioural avoidance and physiological or metabolic resistance. Among the mechanisms involved in physiological resistance, alteration of target site nerve receptors such as *kdr*, *Rdl* and *Ace1.R* have well characterized mutations allowing for efficient assessment of allelic frequencies in local vector populations^10^.

In the Greater Mekong Subregion, malaria cases have significantly decreased and are now mainly confined to pockets of transmission occurring along international borders and in forest or forest fringes^11^. These residual foci of transmission are often populated by ethnic minorities, local populations and rural migrants exploiting new economic opportunities such as rubber plantations, mining and agriculture^11^. In this context, prolonged visits to forest farms increase malaria exposure. Indeed, an overnight stay at the forest plot is one of the main malaria risk factors likely aided by often partially or completely open forest huts as well as variable sleeping time depending on economic activity^12–17^. Since malaria entomological surveys classically collect vectors in villages, only a handful of studies have collected *Anopheles* mosquitoes at forest-fringes or forest sites^16–20^. While a single study found similar mosquito densities at forest and village collection sites when comparing 12 coupled sites in three Cambodian provinces^19^, *Anopheles* mosquito densities are more typically reported as higher in the forest plots compared to the villages^16–18^. Moreover, the number of infected mosquitoes is usually higher in or even exclusive to forest sites^16,18,19^, supporting the increased malaria risk in forest sites.

The complexity of malaria transmission in the Greater Mekong Subregion is further amplified by a high diversity of vector species, including several sibling species only distinguishable molecularly, exhibiting a large spatial heterogeneity in distribution and behavioural patterns both between and within species and species complexes^21–23^. Consequently, a deep understanding of the vectors involved in malaria transmission is extremely difficult without molecular species identifications, even though many studies have relied solely on morphological identifications likely due to the cost involved. Further blurring the pictures of malaria transmission is the tendency to limit malaria parasite screening only to presumptive malaria vectors, and to collections only on humans, or to forego screening altogether^16,24–27^. These short-cuts also tend to underestimate the species involved in malaria transmission in the Greater Mekong Subregion. Nevertheless, on the rare occasions when thorough sampling and screening occurs, the results highlight a high diversity of malaria vectors including presumed zoophilic vectors^28,29^, which is expected since most of the *Anopheles* mosquitoes in the Greater Mekong Subregion often exhibit zoophilic or opportunistic feeding behavior^16,17,25^. In addition, zoophilic preferences are also reported in populations of the main malaria vectors such as *A. dirus* or *A. minimus*^21,23,30–32^. Thus, in an era of malaria elimination in the Greater Mekong Subregion, embedded in a fast-changing environment, limiting screening to anthropophilic and/or classical primary vectors is likely to underestimate the importance of behaviourally opportunistic mosquitoes that could maintain residual malaria transmission, thereby decreasing the effectiveness of malaria elimination programs.

In sampling for malaria vectors, mosquitoes are traditionally collected between the times of 18:00 and 06:00 h, even though Southeast Asian (SEA) malaria vectors are mainly early- and outdoor biters^14,22,33,34^. Nonetheless, some studies extended the collection time, usually between villages and forest sites^18,19^ or in the forest sites^17^ and even sometimes in villages^14^, to better understand human exposure to mosquito bites. Comparison of outdoor collection traps during day and night collections resulted in sampling only a few *Anopheles* during daytime^35^, although a longitudinal study with 24 h collections found most of *A. barbirostris s.l*. mosquitoes collected during daytime^20^. Altogether, these results suggest that longer sampling time of malaria vectors could produce a more comprehensive overview of human exposure to malaria mosquito bites.

Although efficient human disease vectors usually exhibit anthropophilic behavior (i.e. preference for humans as a source of blood) strong spatial, temporal and population variations are observed^36,37^. Host-choice tests using either human or cow odor as alternative attractants allow researchers to assess the inherent host preference of mosquito vector populations. Paired-odor entry traps have been successfully used in the field to study host preferences^37–40^ and comparisons with odor baited double net traps showed similar anthropophily indices^41^. In addition, a study in the Lao PDR confirmed that human-baited double net traps collected similar number of *Anopheles* mosquitoes as the ‘gold standard’ human-landing catch method^35^ which led us to use odor-baited traps to investigate mosquito bionomics in a Cambodian district. We investigated *Anopheles* mosquito bionomics in four ecological settings (villages, forested areas near villages, rubber tree plantations and forest sites) during the 2017 rainy season and the 2017–2018 dry season. Using paired odor-baited traps in a choice arrangement, we assessed odor-mediated mosquito host preference. We explored *Anopheles* activity rhythms by conducting 24 h collections. We screened the collected mosquitoes for carriage of malaria parasites to determine their role in disease transmission. Finally, we complemented morphological identification with DNA sequence-based identification on a subsample of mosquitoes and we investigated mutation patterns in known insecticide resistance genes.

## Methods

### Collection sites

**Figure 1:**
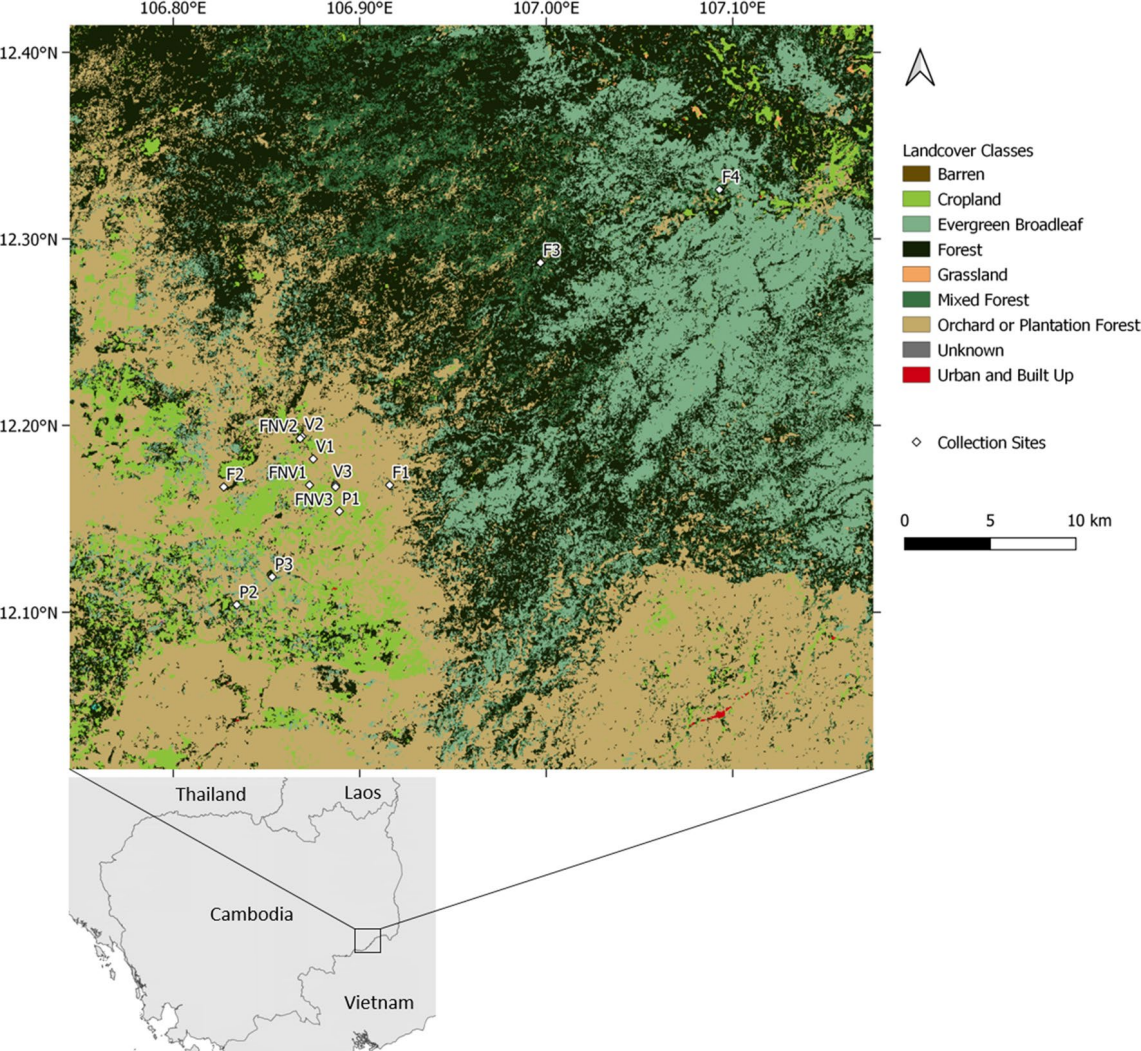
Map of the mosquito collection sites in Kaev Seima district, Mondulkiri, Cambodia. Land use map from Landsat satellite imagery retrieved from SERVIR Mekong (https://landcovermapping.org/en/). Map created in QGIS version 3.14.0 Pi (https://www.qgis.org/en/site/). Collection sites: F = forest site, V = village, FNV = forest area near the village, P = rubber tree plantation.

The study was conducted in Kaev Seima district, Mondulkiri Province, in northeastern Cambodia. This province is characterized by hilly-forested areas and hotspots of ongoing malaria transmission. The main source of income in the district is based on agricultural activity such as subsistence farming and commercial plantations, details on malaria epidemiology were previously published^42^. Mosquito collections were carried out during the rainy season in July–August 2017 and during the dry season in December 2017–January 2018. Four different types of sites were sampled: villages (3 sites), forested areas near the villages (within approximately 200 m from the village, 3 sites), rubber tree plantations (3 sites) and forest sites (4 sites, but one site, initially forest site 3, was changed to forest site 4 during the dry season collection as logistics prevented access to the original site) (Fig. 1). The forest sites were chosen based on interviews with recent *Plasmodium falciparum* symptomatic cases, all males between 14 and 47 years old who had spent time in the forest two weeks before their malaria symptoms.

### Collection trap

Two odor-baited double net traps (BNT) as in Tangena et al.^35^ baited with calf and human odors were used to assess the host preference of field populations of mosquitoes in the villages, forest sites and the forested areas near the villages. The two BNTs consisted of an outer untreated bed net (200 × 200 × 250 cm for the human BNT (HBNT) and 300 × 300 × 300 cm for the cow BNT (CBNT) from which each corner was raised 20 cm above ground and a smaller untreated inner bed net (180 × 180 × 250 cm for the HBNT and 280 × 280 × 280 cm for the CBNT). In the HBNTs, the two human volunteers rested on metal-framed beds (180 × 60 cm) and were protected from mosquito bites by the inner net. HBNTs and CBNTs were operated for 24 h for 2 days in each collection sites and were set up at the same place with the same volunteers. Trapped mosquitoes were retrieved every hour for 10 min using a mouth aspirator. Mosquitoes were transferred to paper cups covered with netting and labelled by date, type of trap, location and hour of collection. Cups were kept with a humid towel on top and brought back every day to the laboratory for further processing (see below). Only HBNTs were set up in the plantation sites to not interfere with rubber tree tapping. Two HBNTs per plantation site were set-up to investigate edge effects.

### Laboratory processing of samples

All *Anopheles* sp. mosquitoes were morphologically identified using a taxonomic key^43^. Following morphological identification, samples were preserved in 70% ethanol until molecular biology analyses. The mosquito heads and thoraxes were crushed individually and DNA was extracted using the QIAamp DNA Mini kit, (Qiagen,Germany), according to the manufacturer’s instructions. A two-step semi quantitative real-time PCR was performed to detect malaria parasites, as previously described^44^.

### Amplicon sequencing

To benchmark the accuracy of our morphological species called, as well as to refine species assignments within morphologically identical taxa, a subset of samples was sequenced to determine mosquito species. To generate the subset, we randomly selected an equal number of samples collected in the CBNTs to match the sample size of the HBNTs (n = 734 samples). Among these 1468 (734 from HBNT and 734 from CBNT) samples we randomly selected a final set of 844 to represent the diversity of trap type, collection site and season. In addition, we used the same sequencing technique to complete the molecular species determination for an additional 79 *Plasmodium sp*. infected samples which were not part of the random selection, bringing to 923 the total number of samples molecularly screened.

### Sequencing of ITS2 for molecular determination of species

Mosquito species was determined molecularly via PCR amplification and Sanger sequencing of the ribosomal DNA internal transcribed spacer region (ITS2; see supplementary information for PCR conditions). All sequence reads were obtained using standard Sanger sequencing methods and resulting sequence traces were visually verified prior to being submitted to nucleotide blast. Resulting sequences were blasted against the NCBI GenBank database. If a given sample gave at least 250 bps of clean sequence and the percent identity of the input ITS2 sequence with a single published (preferably voucher) sequence was 98% or higher, a species determination was made. We were able to confidently assign species for 774 of the 844 samples this way.

In the rare event samples where species determination was not possible (~ 8% of total, 67 samples/841 total samples with species calls) either using ITS2 sequence (n = 2), or obtaining ITS2 sequence was technically problematic (n = 65), amplification and sequencing of the mitochondrial cytochrome oxidase subunit 1 (CO1) gene was carried out using Sanger sequencing with quality control as for ITS2, discussed above (see supplementary information for PCR conditions).Two samples gave high quality ITS2 sequence and high quality CO1 sequence, but did not have a match in the database meeting species assignment parameters.

### Sequencing of known insecticide resistance mutations

Of the 844 samples that were molecularly typed for species, a randomly chosen subset of 192 samples representative of species and collection location were sequenced for the presence of known insecticide resistance mutations for *ace-1* G119S, *rdl* A296S, and *kdr* L1014F (see supplementary information for PCR and sequencing details). These mutations were chosen as they are well described and have been associated with insecticide resistance^45–47^. All sequence reads were obtained using standard Sanger sequencing methods and resulting sequence traces were visually verified prior to examination of known insecticide resistance mutations. All heterozygote calls were verified with a second PCR amplification and sequencing. The G119S mutation within the acetylcholinesterase subunit 1 (*ace-1*) gene has been previously shown to be associated with insecticide resistance^45^. The A296S mutation in exon seven of the *rdl* gene has previously been shown to be associated with dieldrin resistance. The L1014F mutation in the Voltage Gated Sodium Channel (VGSC) gene has been previously shown to be associated with pyrethroid and organochlorine insecticide resistance^47^.

This subset of 192 individuals represented the most abundant species (≥ 20 samples per species) in the original molecular typing of species in 844 samples. To examine insecticide mutation rates in rarer species we also typed the same three mutations in 68 individual samples representing 14 species and 2 unknown anopheline samples.

### Landscape indices

Land use classification over the Keav Seima district was based on Landsat imagery (Landsat 4–8, 30 m resolution, 2017; https://landcovermapping.org/en/). A buffer zone of 2 km around each mosquito collection site was used to determine the percentage of the different land cover classes defined as: cropland, evergreen broadleaf, forests, mixed forests, plantation forests (see supplementary information for land cover class description).

Landscape metrics computation was performed on Fragstats software, version 4.2.1^48^. To reflect landscape diversity and heterogeneity, we computed Shannon’s diversity index (SHDI)^49^, Simpson’s diversity index (SIDI)^50^, patch density (PD) and edge density (ED) at landscape level. SHDI and SIDI allow quantification of diversity (see supplementary information for details), whereas PD and ED indices are fragmentation indices quantifying heterogeneity. In addition, the percentage of landscape of each land cover category was computed at class level.

### Human behaviour indicators

A cross-sectional survey of 4200 inhabitants from Kaev Seima district was carried out between November 2017 and April 2018^42^. A questionnaire was administered to each consenting household members from the randomly selected households. The set of questions regarding sleeping habits, bed nets usage and overnight sleeping in the working areas is summarized below.

### Statistical analyses

*Day biting rate*—We tested the effect of collection site (forest, village, forest near the village, plantation), season (dry *vs*. rainy) and their interactions on the day biting rate (number of *Anopheles* collected between 6 am and 6 pm over the total number of females collected over 24 h) using a General Linearized Mixed Model (GLMM) with a binomial error structure and Site coded as a random factor to account for repeated measurements. Data for the CBNTs and HBNTS were analysed separately.

*Malaria infection rates*—We tested the effect of collection site, season and daytime biting as well as the interaction between collection site and season and the interaction between collection site and daytime on the prevalence of infectious mosquitoes in the CBNTs using a GLMM with a binomial error structure and Site coded as a random factor to account for repeated measurements. The same GLMM was used for the HBNTS without testing for the interactions as an almost complete segregation of the data was observed (no infectious mosquitoes collected in all sites except the forest during the dry season) preventing the model from converging.

*Mosquito host preference*—The anthropophily index (AI) was expressed as the number of *Anopheles sp.* caught in the human-baited trap over the total number of mosquitoes caught in both human- and cow- baited traps. We assessed statistically the anthropophily indices only in the two species having a sufficient number of collected samples as well as a good agreement between morphological and molecular identifications (96.5% and 98.7% agreement for *A. dirus* and *A. kochi* respectively) for meaningful assessment since morphological identifications often encompass several species in the same group. We tested the effect of infection status (uninfected *vs*. infected with the sporozoite transmissible stages), season (dry vs. rainy) and time (day vs. night) on AI using a GLMM with a binomial error structure and Site coded as a random factor.

Frequencies of the molecularly identified species and the insecticide resistance mutations were compared using Chi-square contingency table.

For model selection, we used the stepwise removal of terms, followed by likelihood ratio tests. Term removals that significantly reduced explanatory power (*p* < 0.05) were retained in the minimal adequate model^51^. All analyses were performed in R v.3.0.3. Results are presented as proportion ± 95% confidence interval.

### Ethics statement

For the odor-baited double net traps capture of mosquitoes, the study protocol was reviewed and approved by the National Ethics Committee for Health Research, Ministry of Health, Kingdom of Cambodia (161NECHR). The study procedures, benefits and risks were explained to collectors and their informed consent was obtained. All collectors were followed and symptomatic subjects were administered a standard malaria rapid diagnostic test and referred to a local health care provider for treatment if positive.

The questionnaire from the cross-sectional survey was approved by the National Ethics Committee for Health Research, Ministry of Health, Kingdom of Cambodia (239NECHR) and by the Institut Pasteur Review Board (2017-03). Written informed consent to participate in the study was obtained from all participants or their parent or legal guardian.

All experiments were performed in accordance with relevant guidelines and regulations.

## Results

### Mosquito abundance, biting rate and morphological identifications

A total of 3920 *Anopheles sp.* females, 1167 and 2753 during the dry and rainy seasons respectively, were captured on a total of 60 collection days. Overall 81% (3187/3920) of the samples were collected in the cow odor-baited double net traps (CBNTs) and while this relative abundance was rather consistent between different collection sites for the CBNTs, 67% (490/733) of the *Anopheles* from the human odor-baited double net traps (HBNTs) were collected in the forest sites (Table S1).

The biting rate (# of females/trap/day) for the HBNTs was consistently higher in the forest sites compared to all other locations during both the rainy and the dry seasons (Table 1). However, for the CBNTs, while the biting rate was the highest in the forest sites during the dry season, the tendency changed during the rainy season with a higher biting rate in the villages and the forests near the villages compared to the forest sites (Table 1).

**Table 1:** Biting and infectious rates of *Anopheles* mosquitoes collected by HBNTs and CBNTs across sites and seasons.

Season	Dry		Rainy	
Trap	CBNT	HBNT	CBNT	HBNT
**Forest**				
IR	0.08	0.01	0.03	0.09
BR	96	47	96	35
AI	NA	0.67	NA	3
**Forest near village**				
IR	0.01	0	0.03	0.11
BR	25	5	162	9
AI	NA	0	NA	1
**Plantation**				
IR	NA	0	NA	0.05
BR	NA	3	NA	7
AI	NA	0	NA	0.33
**Village**				
IR	0.01	0	0.01	0.08
BR	15	1	138	6
AI	NA	0	NA	0.5

IR = infection rate (number of infectious mosquitoes/total number of mosquitoes), BR = average biting rate per trap per day, AI = average number of infectious bites per day (IRxBR). NA = non applicable.

A total of 3131 females were morphologically identified as 14 different *Anopheles* species or complexes of morphologically indistinguishable sibling species. Based on these morphological identifications, species thought to be primary vectors comprised only 10.2% of the collected mosquitoes: *Anopheles dirus s.l.* (8.1%, n = 319), *A. minimus s.l.* (0.4%, n = 15) and *A. maculatus s.l.* (1.7%, n = 67). The most abundant species (represented by more than a hundred individuals in our collection) constituted 75.8% of the collected *Anopheles* mosquitoes and were represented by 6 species complexes: *A. barbirostris* (21.2%, n = 831), *A. philippinensis* (14.6%, n = 571), *A. hyrcanus* (13.6%, n = 535), *A. kochi* (10.5%, n = 412), *A. dirus* (8.1%), *A. aconitus* (7.7%, n = 303).

### Molecular determination of mosquito species

A total of 844 females were molecularly characterized for species in the random subset and represent 26 distinct *Anopheles* species as determined by ITS2 and CO1 (Table S2). The most abundant species (representing ≥ 5% of the samples; n ≥ 42) comprise 77.8% of the molecularly typed individuals and represent 8 species from 6 different species complexes. These most abundant species included *A. dirus* (13.2%, n = 112) from the Dirus complex. From the Barbirostris complex; *A. dissidens* (13.2%, n = 112), and *A. campestris-wejchoochotei* (8.1%, n = 69). From the Hyrcanus Group, *A. peditaeniatus* (12.8%, n = 108), and *A. nitidus* (5.7%, n = 48). The Annularis, Funestus, and Kochi Groups were each represented by a single species *A. nivipes* (9.2%, n = 78), *A. aconitus* (6.7%, n = 57), and *A. kochi* (8.7%, n = 74), respectively. The 18 less abundant species, represent by fewer than 42 samples and in many cases just a handful of samples included *A. philippinensis* (n = 17) and *A. annularis* (n = 1) from the Annularis Group, *A. jamesii* (n = 16), *A. pseudojamesi* (n = 1), and *A. splendidus* (n = 1) from the Jamesii Group and *A. saeungae* (n = 29) and *A. barbirostris* (n = 2) from the Barbirostris Group. From the Hyrcanus Group *A. crawfordi* (n = 40), *A. argyropus* (n = 1), *An. nigerrimus* (n = 28), and *A. sinensis* (n = 3) were sampled. *Anopheles maculatus* (n = 22), *A. sawadwongporni* (n = 4), and *A. rampae* (n = 2) from the Maculatus Group. *Anopheles tessellatus* from the Tessellatus Group and *A. interruptus* from the Asiaticus Group were each sampled once. *A. vagus* (n = 12) and *A. karwari* (n = 3) were also present. There were 2 mosquitoes that had 99.9% identical ITS2 and 99.4% identical CO1 sequences but matched no species in the NCBI database. In addition to the random subset, 79 *Plasmodium sp*. infected samples were molecularly characterized for species which resulted in a total of 29 *Anopheles* species as determined by ITS2 and CO1.

### Day biting rate

**Figure 2:**
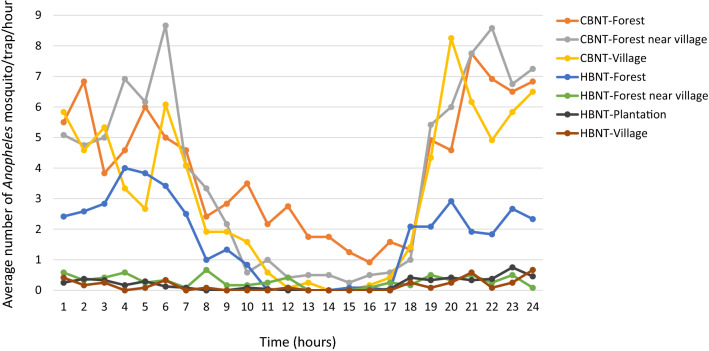
Average number of *Anopheles* females collected per trap per hour in the different collection sites in the HBNTs and the CBNTs.

Overall 20.2 ± 1.2% of the *Anopheles* females were captured during the daytime (between 06:00 and 18:00). Indeed, while the majority of *Anopheles* mosquitoes bite at night, an important proportion was active during the day (Fig. 2). Excluding species with extremely low sample sizes and unidentified samples, day biting behaviour was observed for all the *Anopheles* species and varied from 13 to 68% (Table S3).

**Figure 3:**
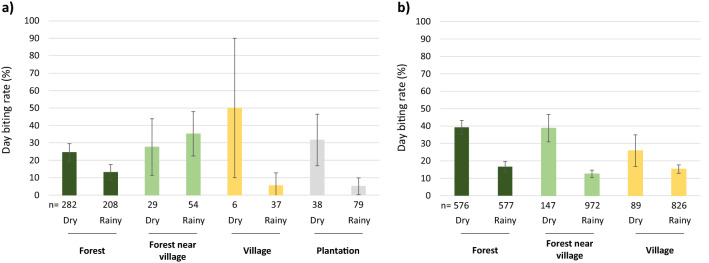
Day biting rate (proportion of *Anopheles* females collected between 6am and 6 pm) of mosquitoes captured in the different collection sites and seasons in (**a**) HBNTs and (**b**) CBNTs. The day biting rate was significantly higher during the dry season compared to the rainy season in both kind of traps (HBNTS χ^2^ = 19.08, df = 1, *p* < 0.001 and CBNTS χ^2^ = 115.53, df = 1, *p* < 0.0001). Data show average proportion ± 95% confidence intervals.

The day biting rate in the HBNTs was not different across collection sites (19.6 ± 2.9%; χ^2^ = 3.6, df = 3, *p* = 0.3; Fig. 3a) but was higher during the dry season (25.9 ± 4.6%) compared to the rainy season (13.8 ± 3.5%; χ^2^ = 19.08, df = 1, *p* < 0.001). There was an overall trend for a higher day biting rate during the dry season compared to the rainy season for all sites except in the forest near the villages where an opposite tendency was observed, however the interaction between collection sites and seasons was not significant (χ^2^ = 7.5, df = 3, *p* = 0.057).

The day biting rate in the CBNTs was higher during the dry season (37.7 ± 3.3%) compared to the rainy season (14.5 ± 1.4%; χ^2^ = 115.53, df = 1, *p* < 0.0001). There was no significant difference across the collection sites (20.4 ± 1.4%; χ^2^ = 0.65, df = 2, *p* = 0.72; Fig. 3b) nor of the interaction between collection sites and seasons (χ^2^ = 1.97, df = 2, *p* = 0.37).

### Malaria infection rates

**Figure 4:**
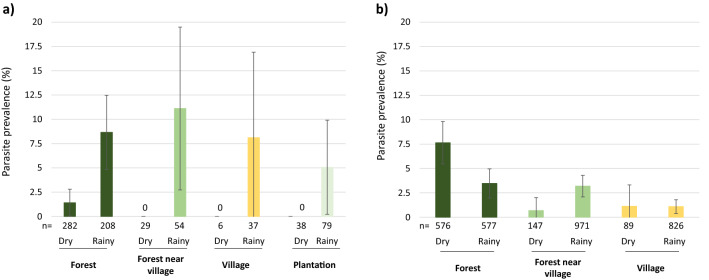
Malaria parasite prevalence in mosquitoes captured across different collection sites and seasons in (**a**) HBNTs and (**b**) CBNTs. Data show average proportion ± 95% confidence intervals. Numbers below the bars indicate the total number of mosquitoes. Zero in the graph indicates no infected mosquitoes. Malaria parasite prevalence was significantly higher during the dry season compared to the rainy season in the HBNTS (χ^2^ = 8.57, df = 1, *p* = 0.003) whereas the opposite was observed in the CBNTS (χ^2^ = 5.44, df = 1, *p* = 0.02). In the CBNTS, malaria parasite prevalence was significantly affected by daytime (χ^2^ = 8.13, df = 1, *p* = 0.004) and the interaction between collection sites and seasons (χ^2^ = 6.94, df = 2, *p* = 0.03).

Samples were screened to detect *Plasmodium sp*. in the head and thorax, thus indicating mosquitoes likely to be infectious due to carriage of sporozoites in the salivary glands (Fig. 4). Over the 3919 mosquitoes tested, 141 positive samples were found (3.6%; 122 *P. vivax*, 13 *P. falciparum*, 4 mixed *P. falciparum* and *P. vivax*, 1 mixed *P. falciparum* and *P. vivax* and *P. ovale*, 1 mixed *P. falciparum* and *P. inui*). In addition, 2 mosquitoes were found infected by *P. cynomologi* and 3 by *P. inui*, these non-human malaria parasites were not included in the following analyses. Malaria infection rate in HBNTs (4.77 ± 1.5%) was not significantly higher than in CBNTs (3.33 ± 0.62%; χ^2^ = 3.19, df = 1, *p* = 0.07). In the HBNTs, malaria infection rate was significantly higher during the rainy season (8.2 ± 2.8%) compared to the dry season (1.13 ± 1.1%; χ^2^ = 8.57, df = 1, *p* = 0.003; Odds Ratio = 5.04 [1.53–16.53]). *Plasmodium sp*. prevalence was not significantly correlated with collection site (χ^2^ = 1.89, df = 3, *p* = 0.60), nor with time of sampling (χ^2^ = 0.22, df = 1, *p* = 0.64).

In the CBNTs, the prevalence of *Plasmodium*-positive mosquitoes was significantly higher during the dry season (5.66 ± 1.6%) compared to the rainy season (2.53 ± 0.6%; χ^2^ = 5.45, df = 1, *p* = 0.02). Malaria infection rate was not significantly different across collection sites (χ^2^ = 5.87, df = 2, *p* = 0.053), although more infectious mosquitoes were collected in the forests (5.55 ± 1.32%) compared to the forest near the villages (2.86 ± 1%) and the villages (1.1 ± 0.7%). Malaria infection rate was higher in mosquitoes collected during daytime (6 ± 1.8%) compared to night time (2.64 ± 0.6%; χ^2^ = 8.13, df = 1, *p* = 0.004) regardless of the collection sites (daytime: collection site interaction: χ^2^ = 3.28, df = 2, *p* = 0.19). Malaria infection rate was significantly influenced by the interaction between collection sites and seasons (χ^2^ = 6.89, df = 2, *p* = 0.03) with opposite patterns in the malaria infection rates observed during the dry and the rainy seasons in the forests and the forest near the villages (χ^2^ = 6.51, df = 1, *p* = 0.032, all other post-hoc comparisons were non-significant; Fig. 4).

The infectious mosquitoes collected in the HBNTs belonged to 8 different mosquito species (molecular identification; Table 2). Interestingly, an additional 12 *Anopheles* species were also found infected by human malaria parasites among the mosquitoes collected in the CBNTs (*A. aconitus*, *A. campestris-wejchoochotei*, *A. crawfordi*, *A. jamesii*, *A. karwari*, *A. minimus*, *A. nivipes*, *A. pallidus*, *A. rampae*, *A. saeungae*, *A. sawadwongporni*, *A. vagus*), bringing to 20 the total number of malaria vectors collected in this study among the 29 species molecularly identified (Table 2).

**Table 2:** Individual *Plasmodium sp*.-positive *Anopheles* specimens collected by HBNTs and CBNTs across sites and seasons.

Trap	Morphological identification	Molecular identification	Number of *Plasmodium sp*. positive samples	Season	Collection site and season
HBNTs	*A. barbirostris*	*A. dissidens*	2	Rainy	Forest near village rainy (2)
		*A. nigerrimus*	1	Rainy	Plantation rainy (1)
	*A. dirus*	*A. dirus*	21	Dry (4), rainy (17)	Forest dry (4), forest rainy (14), forest near village rainy (2), plantation rainy (1)
		*A. nitidus*	1	Rainy	Forest rainy (1)
		Unidentified	2	Rainy	Forest rainy (2)
	*A. hyrcanus*	*A. peditaeniatus*	3	Rainy	Forest near village rainy (1), village rainy (2)
	*A. kochi*	*A. kochi*	1	Rainy	Forest rainy (1)
	*A. maculatus*	*A. maculatus*	1	Rainy	Plantation rainy (1)
	Unidentified	*A. maculatus*	1	Rainy	Plantation rainy (1)
		*A. nitidus*	1	Rainy	Forest near village rainy (1)
		*A. sinensis*	1	Rainy	Village rainy (1)
CBNTs	*A. aconitus*	*A. vagus*	2	Rainy	Village rainy (1), forest near village rainy (1)
		*A. aconitus*	9	Dry	Forest dry (8), village dry (1)
	*A. barbirostris*	*A. campestris—wejchoochotei*	4	Dry	Forest dry (4)
		*A. dissidens*	17	Dry (11), rainy (6)	Forest dry (11), forest rainy (2), forest near Village rainy (3), village rainy (1)
		*A. peditaeniatus*	2	Rainy	Forest near village rainy (2)
		*A. saeungae*	1	Dry	Forest (1)
	*A. dirus*	*A. dirus*	3	Rainy	Forest rainy (3)
	*A. hyrcanus*	*A. aconitus*	1	Rainy	Forest near village rainy (1)
		*A. crawfordi*	6	Dry	Forest dry (6)
		*A. nigerrimus*	4	Dry (1), rainy (3)	Forest near village rainy (1), forest dry (1), forest rainy (2)
		*A. nitidus*	4	Dry (3), rainy (1)	Forest dry (3), forest rainy (1)
		*A. peditaeniatus*	11	Rainy	Forest rainy (3), forest near village rainy (7), village rainy (1)
	*A. insulaeflorum*	*A. peditaeniatus*	2	Rainy	Forest near village rainy (2)
	*A. jamesii*	*A. jamesii*	1	Rainy	Forest near village rainy (1)
		*A. pallidus*	2	Rainy	forest rainy (2)
	*A. kochi*	*A. kochi*	11	Dry (5), rainy (6)	Forest dry (5), forest rainy (5) forest near village rainy (1)
		*A. karwari*	1	Rainy	Forest rainy (1)
	*A. maculatus*	*A. maculatus*	2	Dry (1), rainy (1)	Forest near village dry (1), forest near village rainy (1)
		*A. rampae*	1	Dry	Forest dry (1)
		*A. sawadwongporni*	2	Dry	Forest dry (2)
	*A. philippinensis*	*A. jamesii*	2	Rainy	Forest near village rainy (1), village rainy (1)
		*A. nivipes*	7	Rainy	Forest near village rainy (3), village rainy (4)
	Unidentified	*A. dirus*	1	Rainy	Forest rainy (1)
		*A. dissidens*	1	Rainy	Forest rainy (1)
		*A. minimus*	1	Dry	Forest dry (1)
		*A. nigerrimus*	2	Rainy	Forest near village rainy (2)
		*A. peditaeniatus*	6	Rainy	Forest near village (5), village (1)

Sample sizes are given in parentheses.

### Mosquito host preferences

We assessed the inherent mosquito host preference by calculating the anthropophily index (AI) for the different species, defined as the number of *Anopheles sp.* individuals caught in the human-baited trap over the total number of mosquitoes caught in both human- and calf- baited traps (see Methods). Most of the collected mosquitoes were zoophilic (AI < 50) or generalist (AI ~ 50) including *A. dirus* or *A. maculatus* mosquitoes (Table S4).

*Anopheles dirus*—A total of 319 *An. dirus* females were collected, mainly in the forest sites (n = 303). Human *Plasmodium sp.* prevalence was 8.46 ± 3% overall and 7.9 ± 3% in the forest sites. The anthropophily index was calculated in the forest sites only and was 48.51 ± 5.63%. Infectious mosquitoes were significantly more anthropophilic (87.5 ± 13.23%) than uninfected mosquitoes (45.16 ± 5.84%; χ^2^ = 21.56, df = 1, *p* < 0.0001). *Anopheles dirus* females were more anthropophilic during the dry season (51.83 ± 7.65%) compared to the rainy season (44.6 ± 8.26%; χ^2^ = 7.19, df = 1, *p* = 0.008). There was no difference in the anthropophily index of *A. dirus* collected during the day or night time (χ^2^ = 3.39, df = 1, *p* = 0.066).

*Anopheles kochi*—A total of 412 *A. kochi* females were collected with more than half collected in the forest sites (n = 257). Human *Plasmodium sp.* prevalence was 3.15 ± 1.69% overall and 4.67 ± 2.58% in the forest sites. There was no significant effect of season (χ^2^ = 1.98, df = 1, *p* = 0.16) or infection status (χ^2^ = 0.67, df = 1, *p* = 0.41) on the anthropophily index of *A. kochi*. *Anopheles kochi* females were significantly more anthropophilic during the daytime (25.64 ± 9.69%) compared to night time (8.98 ± 3.07%; χ^2^ = 8.53, df = 1, *p* = 0.003).

**Figure 5:**
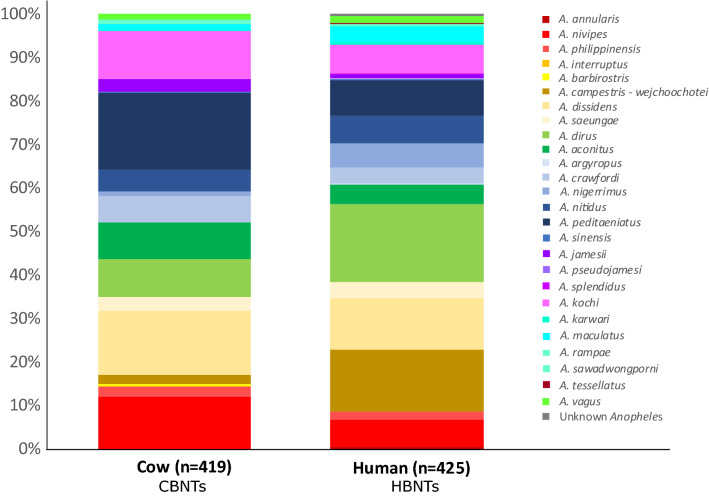
Proportions of the molecularly identified samples in the CBNTS and HBNTS.

Out of the random subset 26 species were molecularly identified, among which 19 species were collected in both HBNTs and CBNTs although in variable proportions (Fig. 5). Indeed, *A. annularis* (n = 2), *A. argyporus* (n = 1), *A. splendidus* (n = 1) and *A. tessellatus* (n = 1) were only observe in the CBNTs, whereas *A. barbirostris* (n = 2), *A. interruptus* (n = 1) and *A. pseudojamesi* (n = 1) were only observed in the HBNTs. Among the 19 species collected in both type of traps, 3 species were enriched in the CBNTs—*A. nivipes* (n = 78, χ^2^ = 8.332, df = 1, *p* = 0.039), *A. peditaeniatus* (n = 108, χ^2^ = 17.334, df = 1, *p* < 0.0001), *A. kochi* (n = 74, χ^2^ = 4.944, df = 1, *p* = 0.026)—whereas 2 species were enriched in the HBNTs—*A. campestris-weichoochetei* (n = 69, χ^2^ = 40.678, df = 1, *p* < 0.0001), *A. dirus* (n = 110, χ^2^ = 16.413, df = 1, *p* < 0.0001).

### Insecticide resistance mutations

192 mosquitoes were genotyped by amplicon sequencing for known mutations associated with resistance to different classes of insecticides: pyrethroids, carbamates, organochlorides, and organophosphates. We genotyped previously characterized mutations in *ace-1, kdr,* and *rdl* genes, G119S^45^, L1014F^45^ and A296S^52^, respectively. Of the 192 individuals, 187 were successfully genotyped for G119S, 189 for L1014F, and 191 for A296S. The few missing genotypes were due to PCR amplification failure. Twenty-seven individuals (14%) carried the G119S mutation, none of which were heterozygotes. Nineteen (10%) individuals carried the L1014F mutation, five of which were heterozygotes. Twenty-nine (15%) individuals carried the A296S mutation, seven of which were heterozygotes. There were four species (of 109 total) that carried resistant mutations. Two *A. dirus* (of 31 total) carried the *rdl* A296S mutation, a single *A. kochi* individual carried the *ace-1* G119S mutation and an additional *A. kochi* individual carried the *rdl* A296S mutation (18 *A. kochi* individuals total). Two *A. nivipes* individuals carried the *rdl* A296S mutation (21 total). Interestingly, a single species, *A. peditaeniatus* was significantly overrepresented for all resistant genotypes (*ace-1*: χ^2^ = 179, df = 1, *p* = 8.01 × 10^–41^, *kdr*: χ^2^ = 132.4, df = 1, *p* = 1.22 × 10^–30^, *rdl*: χ^2^ = 168.1, df = 1, *p* = 1.92 × 10^–38^)*. A. peditaeniatus* accounted for 96% of individuals carrying the *ace-1* mutation (26/27), 100% of individuals with the kdr mutation (19/19), and 83% of individuals with the *rdl* mutation (24/29); despite the fact that it comprised only 13% (26/192) of the sample set genotyped. To gain a better understanding of this apparent enrichment of known insecticide resistance mutations in *A. peditaeniatus*, we returned to the larger sample set (n = 844) and genotyped the additional 82 individuals not included in the representation plates, for a total of 108 genotyped *A. peditaeniatus*. When resistant allele frequencies were compared between the *A. peditaeniatus* in the representation plates (n = 26) and all other *A. peditaenitus* (n = 82), there was no statistically significant difference (*ace-1*: χ^2^ = 1.69, df = 1, *p* = 0.2, *kdr*: χ^2^ = 0.01, df = 1, *p* = 0.91, *rdl*: χ^2^ = 0.78, df = 1, *p* = 0.38), indicating that the representation plates accurately represented the larger dataset.

**Figure 6:**
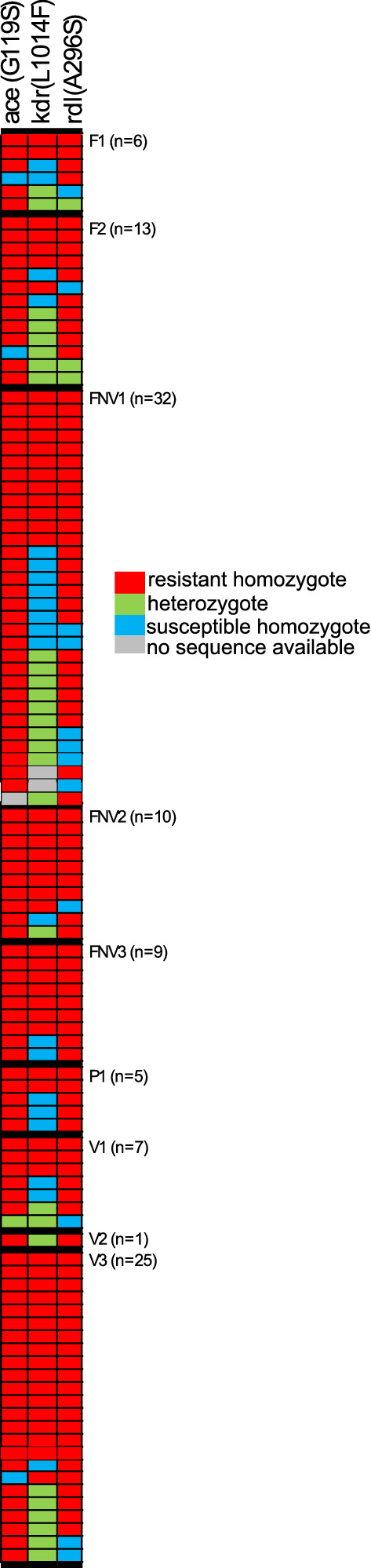
Insecticide resistance mutations are very frequent in *Anopheles peditaeniatus*. All *A. peditaeniatus* (n = 108) in the molecularly typed set of 844 are depicted here sorted by their collection location and within collection site by their insecticide resistance genotypes. Each row of three boxes represents genotype data from a single *A. peditaeniatus* individual. Out of 108 *A. peditaeniatus*, 106 carried the G119S mutation (3 heterozygotes, 103 homozygotes), 78 carried the L1014F mutation (22 heterozygotes, 56 homozygotes), and 104 carried the A296S mutation (12 heterozygotes, 92 homozygotes). Forty nine percent (53/108) of individuals have homozygous resistant genotypes for all 3 loci and an additional 23% (25/108) carry at least one resistant allele for each of the 3 insecticide loci typed. Of the remaining 28% of individuals (30/108), 28 carried the homozygous susceptible allele at the L1014F *kdr* mutation (2 individuals with no available sequence), 17 were homozygous for the resistant allele at both *ace-1* and *rdl*, another 7 individuals were homozygous for *ace* G119S and heterozygous for *rdl* A296S. A single individual was homozygous for the *rdl* resistant mutation and heterozygous for the *ace-1* mutation. Five individuals carried a resistance mutation at only one of the 3 loci tested, 3 were *ace-1* mutation homozygotes, 1 was heterozygous for *rdl* resistance and 1 was homozygous for *rdl* resistance with no sequence available for *ace-1* resistance.

**Figure 7:**
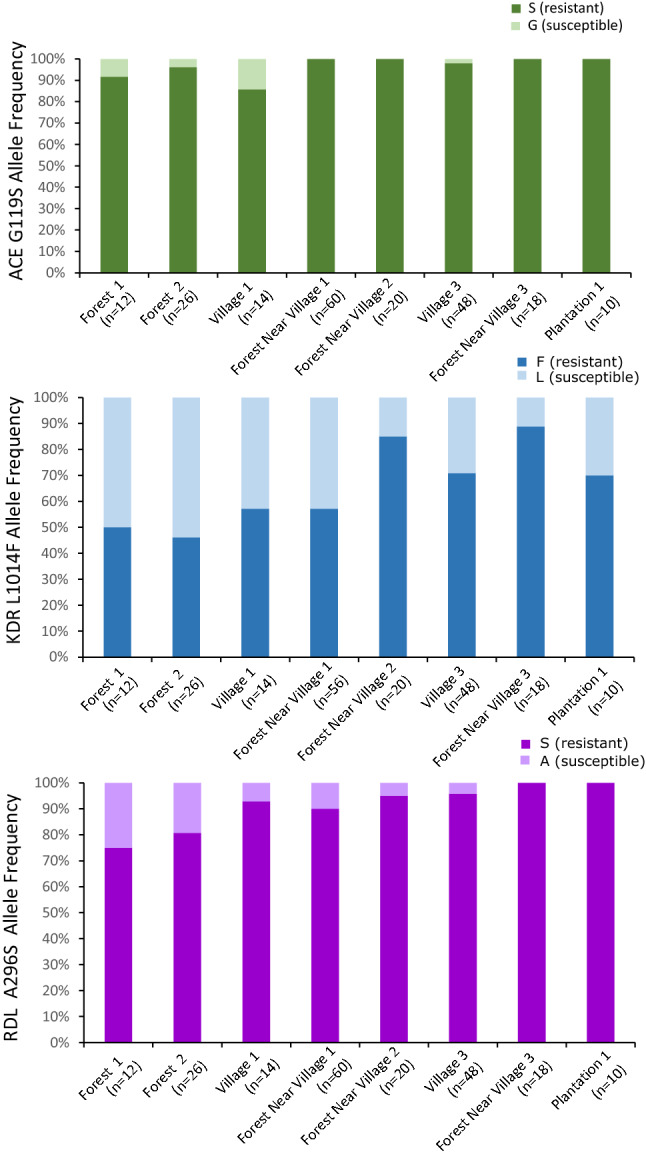
Geographic distribution of insecticide resistance mutations in *A. peditaeniatus. *Allele frequencies for *ace-1* G1119S (top), *kdr* L1014F (middle) and *rdl* A196S (bottom) are shown as a function of sampling location. For *kdr* there is a detectable geographic pattern with the Forest site 2 exhibiting significantly (*p* = 0.03) higher susceptible allele frequency than all other sites combined, while Forest near Village 2 (*p* = 0.04) and 3 (*p* = 0.02) exhibit significantly higher resistant allele frequencies than all other sites combined. A similar pattern is observed for *rdl*, but here the total number of susceptible alleles does not provide the power to detect significant differences. For locations: F = Forest, FNV = Forest Near Village, P = Plantation, and V = Village. Only those 8 sites with at least 5 *A. peditaeniatus* (10 alleles) are shown here. Resistant (res) and susceptible (sus) alleles are colored according to the legends, with resistant alleles in the darker shade.

Of the 108 total *A. peditaeniatus*, 106 carried the *ace-1* G119S mutation (3 heterozygotes; 103 homozygotes), 78 carried the *kdr* L1014F mutation (22 heterozygotes; 56 homozygotes), and 104 carried the *rdl* A296S mutation (12 heterozygotes, 92 homozygotes; Fig. 6). The presence of the *ace-1* mutation, either homozygote or heterozygous was also a strong predictor of whether that individual was also resistant to other insecticides as 97% of individuals that carried the *ace* G119S mutation also had at least one other resistance mutation, homozygote or heterozygote. For the L1014F mutation, the distribution of alleles by ecological site was not uniform (Fig. 7). The L1014F *kdr* resistant allele was significantly more common in the Forests Near Village 2 and 3 (χ^2^ = 4.0, df = 1, *p* = 0.04 and χ^2^ = 5.1, df = 1, *p* = 0.02 respectively) as compared to all other sites. Conversely, Forest 2 had a significantly greater proportion of the susceptible *kdr* L1014F allele (χ^2^ = 4.5, df = 1, *p* = 0.03). The frequency of the *ace-1* G119S mutation was significantly greater than the other mutations (*ace-1 vs kdr*: χ^2^ = 76, df = 1, *p* = 2.76 × 10^−18^, *ace vs rdl*: χ^2^ = 7.8, df = 1, *p* = 5.3 × 10^−3^). Additionally, the frequency of the A296S *rdl* resistant allele was greater than the frequency of the L1014F *kdr* resistant allele (χ^2^ = 45.2, df = 1, *p* = 1.8 × 10^−11^). All heterozygotes were verified by amplifying and resequencing the necessary fragment and visualizing the resultant sequence trace files.

We also examined the frequency of the *kdr* L1014F, *ace-1* G119S and *rdl* A296S insecticide resistance mutations in rarer mosquito species that were detected in the random subset of 844 samples molecularly typed for species. These 68 mosquito samples of rarer species represent 14 species and 2 unknown Anopheline samples (each species represented by 1–17 individuals; median = 2; Supplementary Table S5). In these rare species, rates of resistance alleles were high for *rdl* A296S with resistance being present in 8 of the 14 species and in the unknown Anopheline. In contrast no mosquitoes carried the L1014F mutation in *kdr* and only *A. sinensis* carried the *ace-1* G119S mutation.

### Landscape indices

**Figure 8:**
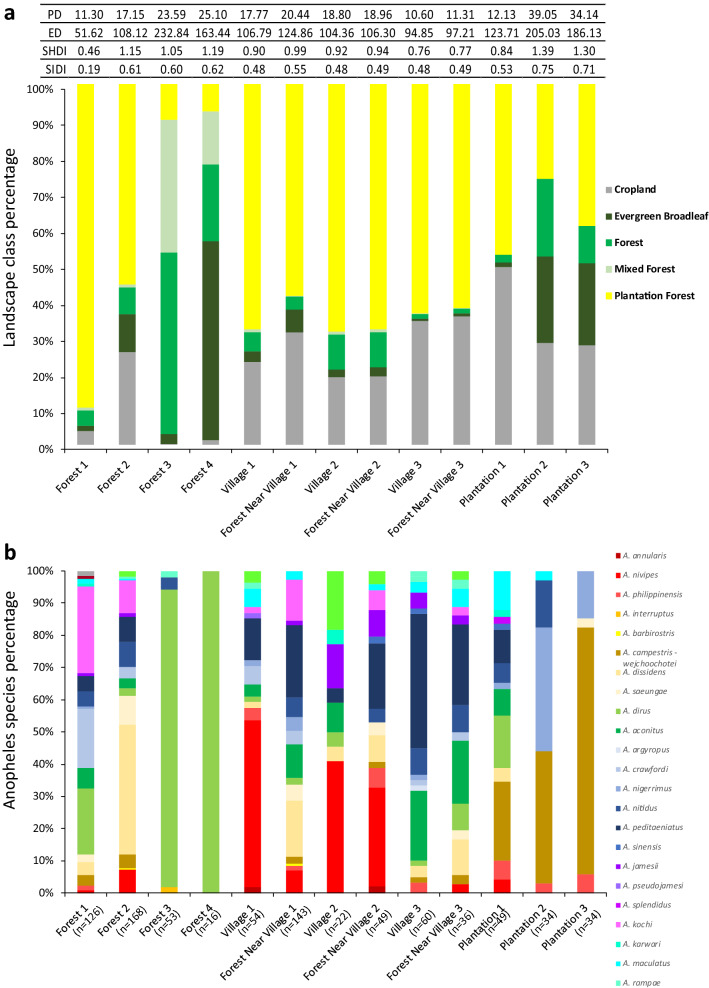
(**a**) Percentage of each land cover category per collection site determined by a 2 km buffer zone and based on Landsat imagery (Landsat 4–8, 30 m resolution, 2017). Land cover classes defined as: cropland (land with herbaceaous and shrubby crops with harvesting and bare soil periods), evergreen broadleaf (land dominated by trees > 60% canopy cover with a tree above 5 m, evergreen broadleaf trees make up > 60% of the total tree cover), forest (land spanning more than 0.5 hectares with trees higher than 5 m and a canopy cover of more than 10%), mixed forests (land with > 60% tree canopy cover, tree height is greater than 5 m, and the forest composition is mixed such that no single forest type makes up > 60% of the total tree cover), plantation forests (land cultivated with perennial crops that reach heights above 5 m and occupy the land for long periods). The table is indicating patch density (PD), edge density (ED), Shannon’s diversity index (SHDI), and Simpson’s diversity index (SIDI) for each buffer zone. (**b**) Percentage of each mosquito species (molecular identification) sampled in each collection site. Sample sizes are given in parentheses.

A 2 km buffer zone around each mosquito collection site was used to determine the percentage of the different land cover classes present and showed high variability between sites (Fig. 8a). Patches of evergreen forest, forest and plantation forest were observed in all sites. Croplands were observed in all buffer zones, from 0.16% in Forest 3 up to 40.2% in Plantation 1, however the largest human-made land cover class was represented by plantation forests, from 7.5% in Forest 4–89.7% in Forest 1. While more *A. dirus* were observed in Forests 3 and 4 (n = 122 and n = 92 respectively), it was still present in the more degraded landscapes of Forests 1 and 2 (n = 71 and n = 18). The most conserved forest sites (Forests 3 and 4) were also associated with lower densities and lower diversities of mosquitoes as also observed in Plantations 2 and 3 (Fig. 8b).

Although Forests 3 and 4 had high proportions of natural forests, their high PD and ED values indicate more fragmented landscapes than Forests 1 and 2. Patch richness only varies between 4 and 5 types of land cover classes therefore an increase value of SHDI and SIDI corresponded to an increase evenness between patch types, with the highest patch evenness observed in Forests 2, 3 and 4 as well as Plantations 2 and 3. Villages and forest near the villages exhibited uneven patch distribution associated with fragmented landscapes.

### Human behaviour indicators

More than half of the villagers reported going to bed between 19:00 and 20:00 pm and using a bed net the previous night (92%; Table S6). However, only 41% were insecticide-treated bed nets. More than three quarters of the villagers were working in cashew plantations and cassava fields at the time of the study (77%, Table S6). Interestingly, sleeping overnight in the field sites was reported in 3.7–7.81% of the cases, with a vast majority reporting the use of bed nets, whereas 77.9% of people working in the forest sites also slept there with only 25% of them using a bednet (Table S6).

## Discussion

This mosquito collection across ecological zones in northern Cambodian shows a high *Anopheles* mosquito diversity with morphological identification of 14 species complexes and 29 species in the subset of genotyped and molecularly identified mosquitoes (n = 923). A recent study in Cambodia found similarly high mosquito diversity although the 27 species were collected over three different provinces^28^. Three species were unique to the latter collection while 5 other species were unique to our collection, further highlighting the high *Anopheles* mosquito diversity in Cambodia. Here, despite sampling from only 13 sites across a 25 × 30 km area in Mondulkiri district, our results indicate both high mosquito diversity as well as highly variable mosquito densities. Although the sites chosen for mosquito sampling were in close proximity (average distance between any two sites = 10.8 km, min = 0.011 km, max = 28.7 km) especially for the 3 pairs of villages and the forest near the village samples (0.011 km, 0.015 km and 1.56 km), large variations in mosquito species abundance can be observed on a microscale (Fig. 8a). *Anopheles dirus* mosquitoes were collected even in highly degraded and fragmented forest sites, suggesting that they can adapt to human-altered habitats. Interestingly, mosquito densities were similar in the dry and the rainy seasons in the forest sites whereas densities strongly dropped in the dry season in the other sampled sites. Thus, patches of forests might represent refuges for the mosquito populations during the dry season until they disperse again to the field and villages in the rainy season, as has been suggested previously for *A. minimus*^53^ or *A. dirus*^54^. Consequently, these forest refuges might not only maintain *Anopheles* populations, but also serve as a transmission reservoir during the dry season since human activity is also high in the forest sites during this period. This is in line with the local epidemiology where although malaria cases exhibit a seasonal pattern in Cambodia, symptomatic cases are still presenting at the health centers during the dry season.

A higher proportion of mosquitoes were collected in CBNTs as compared to HBNTs (81% of the total collection caught on CBNT) and screening them for infection with malaria parasites added 12 species to the 8 mosquito species already found infected in the HBNTs, for a total of 20 of the 29 species molecularly identified having at least one individual positive for malaria parasites. The efficiency of cow baited traps, either collecting resting mosquitoes from the interior of a surrounding tent or directly on the surrounding vegetation, in collecting large number of mosquitoes has been shown previously^28,29^. Historically the CBNT sampling technique has been dismissed as unrepresentative of malaria parasite vectors. Here, we investigated the innate host-preference of mosquitoes using exactly the same trapping techniques for both human and cow. We showed that the collected species of mosquitoes were all generalist or zoophilic in their preferences. The random subset of molecularly identified species showed that among the 19 species collected in both type of traps, *A. nivipes*, *A. peditaeniatus* and *A. kochi* were enriched in the CBNTs whereas *A. campestris-wejchoochotei* and *A. dirus* were enriched in the HBNTs. These 5 species enriched in one type of trap represented 47 and 25 samples for the CBNTs and the HBNTs respectively, out of the 139 infected samples which were typed (Table 2). Furthermore, the overall malaria parasite prevalence in HBNTs (4.77 ± 1.5%) and CBNTs (3.33 ± 0.6%) were not significantly different with the CBNTs still collecting an important proportion of infectious mosquitoes (75%) represented by 19 different *Anopheles* species. Moreover, as CBNTs are a more efficient means of capturing mosquitoes, a greater numbers of species of mosquitoes are able to be studied and the risk to human volunteers is eliminated. Overall, these results are of importance for two reasons. First, screening for malaria parasites only in mosquitoes collected on humans is likely to miss species involved in malaria parasite transmission unless very large collection efforts are carried out. Second, it suggests that many of these mosquito populations are largely maintained by feeding on animals, and that, different from African malaria vectors, the notion of highly preferential human feeding may be inaccurate in the SEA region. Knowing that opportunistic feeding is very common could inform the design of new vector control tools to control malaria infection levels. Labeling of a particular mosquito species as zoophilic or anthropophilic does not represent the reality of more opportunistic feeding preferences.

We found a total of 20 different *Anopheles* species infected with malaria parasites (Table 2). St. Laurent et al^26^ found 5 different species infected with *P. falciparum* and together both studies identify a total of 22 different *Anopheles* species being able to transmit malaria parasites in Cambodia. These two studies are in strong contrast with another collection in Cambodia which found malaria parasites in only three mosquito species: *A. dirus s.s*., *A. minimus s.s*. and *An. barbirostris s.s.*^19^. However it should be noted that only human landing catches were used in Durnez et al.^17^, whereas all the infected mosquitoes collected in St. Laurent et al^26^ were captured in cow baited tents and 75.2% of infective samples in this study were collected in CBNTs, further highlighting the importance of opportunistic feeding behaviour in SEA mosquitoes. In addition, high malaria parasite prevalence was observed in the local human population (8.3%) with important heterogeneity exemplified by malaria infection prevalence rates of 5.7%, 6.5% and 13.1% in the three villages corresponding to the mosquito collection sites^42^. These persisting pockets of high malaria risk as well as our selection of forest sites based on recent *P. falciparum* cases are likely to explain the high malaria parasite prevalence as well as the high diversity of infected mosquitoes observed compared to previous collections.

*Anopheles dirus* and *Anopheles minimus* are usually considered the main malaria vectors in the Greater Mekong Subregion^21,22^. Whereas *A. dirus* represented 8.1% (n = 319) of the collected mosquitoes in this study and were concentrated in forest sites (n = 303), very few *A. minimus* (0.4%, n = 15) were captured in our study. The collection carried out in 2013 by St Laurent et al^28^ found less than 2% of *A dirus* (n = 29) and *A. minimus* (n = 7) mosquitoes. Interestingly, the work from Durnez et al^19^ carried out in 2005 in three Cambodian provinces found 10.3% of *A. dirus* mosquitoes and 24.8% of *A. minimus s.l*. with a majority being *A. minimus s.s.*. Several hypotheses could explain these differences in species composition. First, collections using cow baited traps in our study and the study by St. Laurent and colleagues likely increased species diversity and numbers, consequently decreasing individual species relative abundance. Secondly, variability between sampling sites is likely to be an important factor influencing the relative abundance of mosquito species collected. Thirdly, the increased human influence upon Cambodian landscapes including the studied area^55–57^ is likely to impact mosquito ecology over time and space resulting in large changes in *Anopheles* communities. Finally, malaria vector control measures might have shifted the *Anopheles* species involved in malaria parasite transmission as observed in Africa^58,59^.

It is generally accepted that the vast majority of mosquito feeding happens in the period between dusk and dawn. Despite this, we collected ~ 20% of *Anopheles* mosquito between 6 am and 6 pm, with light conditions in the forest which might resemble dusk or night conditions and might explain the higher density of females collected in this environment (Fig. 2). Malaria vectors are traditionally collected between 6 pm and 6 am since these 12 h usually encompass their peak biting times. Unlike their African counterparts, SEA *Anopheles* are known to be mainly early-biters and extended collections have sometimes been carried out^14,17–19^, with two studies sampling mosquitoes over 24 h^20,35^. Although these studies did collect small numbers of *Anopheles* during the day, the overall sample sizes were considerably lower than those we report here^20,35^. The non-negligible proportion of *Anopheles* mosquito biting during the daytime, when people are active and not protected by a bed net, highlights the importance of residual transmission in this area. Recent estimates of residual transmission in Africa found that between 5 and 40% of mosquito bites occur when people are out and not protected by a bed net^60^. Similar estimates would be needed to adequately quantify the importance of residual transmission in SEA, still, with local vectors being mainly early and outdoor -biters, SEA estimates are likely to be at least equal if not largely higher than those reported for Africa.

Malaria infection rate in the HBNTs was not significantly correlated with the different collection sites, which is likely due to the comparatively low numbers of mosquitoes sampled from villages, forest near the villages and plantations as compared to the forest sites as well as the absence of infectious mosquitoes collected in these sites during the dry season (Fig. 4). There were 6 times more infectious bites per day in the forests compared to the villages during the rainy season (Table 1) further supporting the higher malaria risk in forests in at least northern Cambodia. Few people reported sleeping overnight when working in their field or plantation sites (3.7–18.9%) and when they did, most of them used bed nets (89–100%; Table S6). However, an opposite tendency was observed in the forest sites, with ~ 78% reporting sleeping overnight in the forest when working there with only a quarter of them using a bed net (Table S6). Coupled with the fact that a non-negligible proportion of vectors bite during the day time, these observations call for new malaria vector control tools adapted to forest work activities. These control tools would ideally be easy to use and carry, and provide protection during both day and night hours.

The random subset of mosquitoes genotyped for known insecticide resistance mutations (*ace-1* G119S, *rdl* S196, and *kdr* L1014F) indicated that individuals carried mutations associated with response to all the different classes of insecticides. Indeed, among the 192 mosquitoes genotyped only 4 out of the 12 species represented were found carrying any of the three genotyped insecticide resistance mutations: *A. dirus*, *A. kochi*, *A. nivipes* and *A. peditaeniatus*. Interestingly, two *A. dirus*, two *A. nivipes* and one *A. kochi* individuals had an *rdl* mutation associated with dieldrin resistance, one *A. kochi* carried an *ace-1* mutation associated with carbamate and organophosphate cross-resistances, while all other mutations were observed in *A. peditaeniatus* (26 *ace-1* mutations, 19 *kdr* mutations and 24 *rdl* mutations in 26 *A. peditaeniatus*). Since *A. dirus* is considered a forest mosquito, insecticide exposure and thus development of resistance may be less likely, although potential resistance to DDT was noted at one site in Cambodia^61^. However, with fragmentation and degradation of forests, human activities are increasingly in closer contact with sites harbouring forest mosquitoes leading to potential insecticide resistance emergence even in forest-associated species. Interestingly, insecticide resistance genotypes were overrepresented in *A. peditaeniatus* (Fig. 6). This mosquito species is mainly zoophilic, outdoor biting and breeding in ponds and rice fields^29,62–66^. Therefore exposure to agricultural pesticides could explain the observed pattern of insecticide resistance mutations. Even though *A. peditaeniatus* is not considered a primary malaria vector due to its zoophilic behaviour, several occurrences, including this collection, of *Plasmodium sp*. infection have been found^28,29,67–69^. In addition, entomological collections during a malaria outbreak in Thailand found a very high prevalence of this mosquito species, with ~ half of the tested individuals being resistant to at least one insecticide class, further suggesting a potential involvement of *A. peditaeniatus* in malaria transmission^46^. Thus, *A. peditaeniatus* might play a more important role than suspected in maintaining residual malaria transmission and knowledge of its insecticide resistance mutations is important as malaria control tools are optimized.

Overall, this study illustrates the importance of diverse collection methods, sites and seasons to better characterize *Anopheles* mosquito ecology in SEA. In addition, the increased patchiness and landscape changes following economy-driven human activities is likely to result in large changes in *Anopheles* communities over time and space. The frequency of opportunistic feeding and day-biting behavior highlights key mechanisms driving residual human malaria transmission in Cambodia. Further investigations on the importance of day-biting in various *Anopheles* species and ecological sites would be interesting to better quantify its frequency. Finally, the low frequency of known insecticide resistance mutations with the exception of a single vector species suggest that new integrated vector control methods could be added as a component in the malaria vector control toolbox, which would help accelerate malaria elimination in the Greater Mekong sub region. Nevertheless, any increase in insecticide usage should be carefully monitored by frequent measurement of insecticide resistance mutations and mosquito phenotypic assays.

## Supplementary Information

Supplementary Information 1.

Supplementary Information 2.

## Data Availability

Novel Anopheline sequence has been submitted to Genbank with accession numbers https://www.ncbi.nlm.nih.gov/nuccore/MW237766 and https://www.ncbi.nlm.nih.gov/nuccore/MW307220. The dataset generated during the current study is included in supplementary material.
