# Publisher Correction: Anopheles ecology, genetics and malaria transmission in northern Cambodia

**DOI:** 10.1038/s41598-021-91552-1

**Published:** 2021-06-09

**Authors:** Amélie Vantaux, Michelle M. Riehle, Eakpor Piv, Elise J. Farley, Sophy Chy, Saorin Kim, Anneli G. Corbett, Rachel L. Fehrman, Anais Pepey, Karin Eiglmeier, Dysoley Lek, Sovannaroth Siv, Ivo Mueller, Kenneth D. Vernick, Benoit Witkowski

**Affiliations:** 1 Malaria Molecular Epidemiology Unit, Institut Pasteur du Cambodge, Phnom Penh, Cambodia; 2 Department of Microbiology and Immunology, Medical College of Wisconsin, Milwaukee, WI USA; 3 Unit of Insect Vector Genetics and Genomics, Department of Parasites and Insect Vectors, Institut Pasteur, Paris, France; 4 CNRS Unit of Evolutionary Genomics, Modeling, and Health (UMR2000), Institut Pasteur, Paris, France; 5 National Center for Parasitology, Entomology and Malaria Control Program, Phnom Penh, Cambodia; 6 School of Public Health, National Institute of Public Health, Phnom Penh, Cambodia; 7 Malaria: Parasites and Hosts Unit, Institut Pasteur, Paris, France

Correction to: *Scientific Reports* https://doi.org/10.1038/s41598-021-85628-1, published online 19 March 2021

The original version of this Article contained an error in Figure 5 where the labels in the legend were incomplete. The original Figure 5 and accompanying legend appear below.

**Figure 5:**
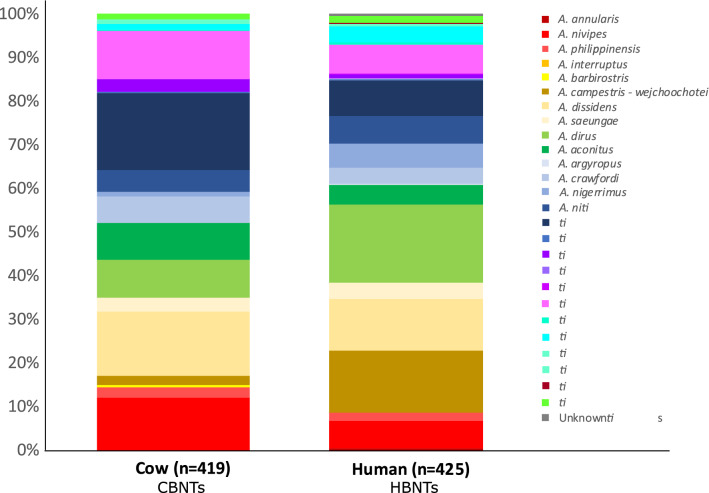
Proportions of the molecularly identified samples in the CBNTS and HBNTS.

The original Article has been corrected.

