# Supplementary material for: Anopheles ecology, genetics and malaria transmission in northern Cambodia
Source: Sci Rep. 2021 Mar 19;11:6458. doi: 10.1038/s41598-021-85628-1 (PMC7979810; doi:10.1038/s41598-021-85628-1)
Supplement: Supplementary file 1 — Supplementary Information 1. [file 41598_2021_85628_MOESM1_ESM.docx]

**Anopheles ecology, genetics and malaria transmission in northern Cambodia**

Amélie Vantaux^1*§^, Michelle M. Riehle^2*^, Eakpor Piv^1^, Elise J. Farley^2^, Sophy Chy^1^, Saorin Kim^1^, Anneli G. Corbett^2^, Rachel L. Fehrman^2^, Anais Pepey^1^, Karin Eiglmeier ^3,4^, Dysoley Lek^5,6^, Sovannaroth Siv^5^, Ivo Mueller^7^, Kenneth D. Vernick^3,4#^ and Benoit Witkowski^1#^

**Methods**

***Sequencing of ITS2 for molecular determination of species***

Amplification of the ITS2 region was performed using the primer pair ITS2A 5’ TGTGAACTGCAGGACACAT and ITS2B 5’ – TATGCTTAAATTCAGGGGGT. The 20 µl PCR reaction contained 5-10ng of DNA template, 0.2µM of each primer and 1X PCRUltramix (PCRBiosystems, UK) (1X PCR buffer, 1mM dNTPs, 3mM MgCl_2_ , and 0.075 U/µl DNA Taq DNA Polymerase). The cycling conditions included an initial denaturation at 94°C for 5 minutes followed by 25 cycles of 94°C for 30 s, 52°C for 30s, and 72°C for 1 minute, and a final extension at 72°C for 5 minutes. PCR products were visualized on 1.5% agarose gels with bands ranging from 430bp-1,860bp depending on the species. In the event of weak or absent bands, PCR cycling conditions from other published work were used. PCR amplicons were enzymatically cleaned prior to sequencing with the addition of 1U Shrimp Alkaline Phosphatase and 1U Exonuclease 1 to 50-100ng of PCR product. This mixture was incubated at 37°C for 15 minutes followed by 80°C for 15 minutes. A minimum of 50 ng of cleaned product was sent for sequencing by MCLab (https://www.mclab.com/).

***Sequencing of CO1 for molecular determination of species***

The 20 µl PCR reaction contained 5-10ng of DNA template, 0.5 uM of each primer (HCO 5' TAAACTTCAGGTGACCAAAAAATCA and LCO 5' GGTCAACAAATCATAAAGATATTGG) and 1X PCR Ultramix (PCRBiosystems,UK) (1X PCR buffer, 1mM dNTPs, 3mM MgCl_2_ , and 0.075 U DNA polymerase). The cycling conditions included an initial denaturation at 94°C for 5 minutes followed by 5 cycles of 94°C for 40 s, 45°C for 1 minute, and 72°C for 1 minute, followed by another 40 cycles of 94°C for 40 s, 51°C for 1 minute, and 72°C for 1 minute, with a final extension at 72°C for 10 minutes. PCR products of expected size (670bp) were cleaned, sent for sequencing, and analyzed as for ITS2, described above.

***Sequencing of known insecticide resistance mutations***

***Ace-1***

Amplification of the *ace-1* gene was performed using the primer pair AceF 5’ GTGCGACCATGTGGAACC-3’ and aceBack2 5’ – GCATTGCCCGGCGCTTCCGG. The 20 µl PCR reaction contained 5-10ng of DNA template, 0.5 µM of each primer in 1X PCR Ultramix (PCRBIO, UK, containing; 1X PCR buffer, 1mM dNTPs, 3mM MgCl2, and 0.075 U/µl Taq DNA Polymerase). The cycling conditions were 94°C for 5 minutes followed by 32 cycles of 94°C for 1 minute, 58°C for 30s, and 72°C for 1 minute, and a final extension at 72°C for 10 minutes. PCR products of expected size (261bp) were enzymatically cleaned as described above for ITS2. A minimum of 50 ng of cleaned product was sent for sequencing by a MCLab (<https://www.mclab.com/>). All traces were visually examined to make genotype calls. All heterozygote calls were verified by independent PCR amplification and sequencing.

***Rdl***

The 20 µl PCR reaction reaction contained 5-10ng of DNA template, 0.5 uM of each primer in 1X PCR Ultramix (PCRBIO, UK, containing; 1X PCR buffer, 1mM dNTPs, 3mM MgCl2, and 0.075 U/ul Taq DNA Polymerase). Primers were RDLF 5’ AGTTTGTACGTTCGATGGGTTA and RDLR, 5’ CCAGCAGACTGGCAAATACC. The cycling conditions included one cycle at 95°C for 5 minutes followed by 35 cycles of 94°C for 30s, 57°C for 30s, and 72°C for 45 seconds and a final extension for 10 minutes at 72°C. PCR products were visualized, enzymatically cleaned and sequenced as described above. Again, sequence traces were visually examined to genotype the A296S codon.

***kdr***

Amplification of the *kdr* portion of VGC was performed using the primer pair Ag-F kdr 5’ GACCATGATCTGCCAAGATGGAAT-3’ and An-kdr-R2 5’ – GAGGATGAACCGAAATTGGAC-3’. The 20 µl PCR reaction contained 5-10ng of DNA template, 1X PCR buffer, 1mM dNTPs, 3mM MgCl2, 0.5 uM of each primer and 0.075 u/ul DNA Taq DNA Polymerase. The cycling conditions included one cycle at 94°C for 5 minutes, 45°C for 30s, and 72°C for 1 minute and 30 s, followed by 34 cycles of 94°C for 30s, 55°C for 30s, and 72°C for 45s. PCR products were visualized on 1.5% agarose for bands of expected size (300 bp). PCR amplicons were enzymatically cleaned prior to sequencing as described above. Resulting sequence traces were visually examined and the genotypes determined for L1014F.

***Landscape indices***

Land cover classes description: cropland (land with herbaceaous and shrubby crops with harvesting and bare soil periods), evergreen broadleaf (land dominated by tress >60% canopy cover with a tree above 5m, evergreen broadleaf trees make up >60% of the total tree cover), forest (land spanning more than 0.5 hectares with trees higher than 5 m and a canopy cover of more than 10%), mixed forests (land with >60% tree canopy cover, tree height is greater than 5 m, and the forest composition is mixed such that no single forest type makes up >60% of the total tree cover), plantation forests (land cultivated with perennial crops that reach heights above 5 m and occupy the land for long periods).

SHDI increases as the number of different patch types (*i.e.*, patch richness) increases and/or the proportional distribution of area among patch types becomes more even. The SIDI value represents the probability that any two cells selected at random would be different. Thus, the higher the SIDI value the greater the likelihood that any two randomly drawn cells would have different values. Because SIDI is a probability, it can be interpreted in both absolute and relative terms. SIDI reaches 1 as the number of different patch types (*i.e.*, patch richness) increases and the proportional distribution of area among patch types becomes more equitable ^1^.

**Supplementary tables**

Table S1. Total number (n), mean biting rate (BR = number of mosquito per trap per day) and relative abundance of the anopheles mosquitoes collected in the different collection sites and traps.

|  | **CBNT** | | |  | **HBNT** | | |  | **Overall** | | |
| --- | --- | --- | --- | --- | --- | --- | --- | --- | --- | --- | --- |
|  | n | BR | relative abundance |  | n | BR | relative abundance |  | n | BR | relative abundance |
| **Forest** | 1153 | 96 | 36 |  | 490 | 41 | 67 |  | 1643 | 68 | 42 |
| **Forest Near Village** | 1119 | 93 | 35 |  | 83 | 7 | 11 |  | 1202 | 50 | 31 |
| **Plantation** |  |  |  |  | 117 | 5 | 16 |  | 117 | 5 | 3 |
| **Village** | 915 | 76 | 29 |  | 43 | 4 | 6 |  | 958 | 40 | 24 |
| **Total** | 3187 | 89 | 100 |  | 733 | 12 | 100 |  | 3920 | 41 | 100 |

Table S2. Molecular species identification for 844 samples.

| **Molecular Identification** | **Forest** | **Forest Near Village** | **Plantation** | **Village** | **Total** |
| --- | --- | --- | --- | --- | --- |
| **Annularis Group** |  |  |  |  |  |
| *A. annularis* |  | 1 |  | 1 | 2 |
| *A. nivipes* | 13 | 26 | 2 | 37 | 78 |
| *A. philippinensis* | 2 | 5 | 6 | 4 | 17 |
| **Asiaticus Group** |  |  |  |  |  |
| *A. interruptus* | 1 |  |  |  | 1 |
| **Barbirostris Group** |  |  |  |  |  |
| *A. barbirostris* | 1 | 1 |  |  | 2 |
| *A. campestris - wejchoochotei* | 11 | 5 | 52 | 1 | 69 |
| *A. dissidens (barbirostris clade III)* | 73 | 33 | 2 | 4 | 112 |
| *A. saeungae(barbirostris clade IV)* | 18 | 10 | 1 |  | 29 |
| **Leucosphyrus Group** |  |  |  |  |  |
| *A. dirus* | 95 | 6 | 8 | 3 | 112 |
| **Funestus Group** |  |  |  |  |  |
| *A. aconitus* | 13 | 22 | 4 | 17 | 56 |
| **Hyrcanus Group** |  |  |  |  |  |
| *A. argyropus* |  |  |  | 1 | 1 |
| *A. crawfordi* | 29 | 7 |  | 4 | 40 |
| *A. nigerrimus* | 1 | 6 | 19 | 2 | 28 |
| *A. nitidus* | 21 | 14 | 8 | 5 | 48 |
| *A. peditaeniatus* | 19 | 51 | 5 | 33 | 108 |
| *A. sinensis* |  | 1 | 1 | 1 | 3 |
| **Jamesii Group** |  |  |  |  |  |
| *A. jamesii* | 3 | 7 |  | 6 | 16 |
| *A. pseudojamesi* |  |  |  | 1 | 1 |
| *A. splendidus* |  |  | 1 |  | 1 |
| **Kochi Group** |  |  |  |  |  |
| *A. kochi* | 51 | 22 |  | 1 | 74 |
| **Maculatus Group** |  |  |  |  |  |
| *A. maculatus* | 3 | 7 | 7 | 5 | 22 |
| *A. rampae* |  |  |  | 2 | 2 |
| *A. sawadwongporni* | 2 | 1 |  | 1 | 4 |
| **Tessellatus Group** |  |  |  |  |  |
| *A. tessellatus* | 1 |  |  |  | 1 |
| **Other** |  |  |  |  |  |
| *A. karwari* | 1 |  | 1 | 1 | 3 |
| *A. vagus* | 3 | 3 |  | 6 | 12 |
| Unknown *Anopheles* | 2 |  |  |  | 2 |
| **Total** | 363 | 228 | 117 | 136 | 844 |

Table S3. Day biting rate (% of individuals collected between 6 am and 6pm) of the different anopheles species (morphological identification).

| **Species** | **n** | **Day biting rate** |
| --- | --- | --- |
| *A. aconitus* | 303 | 34.98 |
| *A. barbirostris* | 831 | 25.63 |
| *A. dirus* | 319 | 12.85 |
| *A. hyrcanus* | 535 | 24.86 |
| *A. insulaeflorum* | 18 | 11.11 |
| *A. interruptus* | 2 | 50.00 |
| *A. jamesii* | 43 | 20.93 |
| *A. karwari* | 13 | 15.38 |
| *A. kochi* | 412 | 18.93 |
| *A. maculatus* | 67 | 25.37 |
| *A. minimus* | 15 | 66.67 |
| *A. philippinensis* | 571 | 17.51 |
| *A. stephensi* | 1 | 100 |
| *A. tessellatus* | 1 | 0 |
| unidentified | 789 | 10.27 |

Table S4. Total number of individuals collected in the cow and the human baited traps by anopheles species (morphological identification), collection site and season. The anthropophily index (AI) is expressed as the number of *Anopheles sp.* caught in the human-baited trap over the total number of mosquitoes caught in both human- and calf- baited traps.

|  |  | **Dry season** | | | |  | **Rainy season** | | | |
| --- | --- | --- | --- | --- | --- | --- | --- | --- | --- | --- |
|  |  | **Cow** | **Human** | **AI** | **total** |  | **Cow** | **Human** | **AI** | **total** |
| **Forest** |  |  |  |  |  |  |  |  |  |  |
|  | *A. aconitus* | 63 | 23 | 26.74 | 86 |  | 17 | 4 | 19.05 | 21 |
|  | *A. barbirostris* | 150 | 99 | 39.76 | 249 |  | 184 | 52 | 22.03 | 236 |
|  | *A. dirus* | 79 | 85 | 51.83 | 164 |  | 77 | 62 | 44.60 | 139 |
|  | *A. hyrcanus* | 135 | 40 | 22.86 | 175 |  | 64 | 3 | 4.48 | 67 |
|  | *A. interruptus* |  |  |  |  |  | 2 |  | 0 | 2 |
|  | *A. jamesii* | 6 | 2 | 25 | 8 |  | 14 | 1 | 6.67 | 15 |
|  | *A. karwari* | 1 |  | 0 | 1 |  |  | 1 | 100 | 1 |
|  | *A. kochi* | 106 | 27 | 20.30 | 133 |  | 109 | 15 | 12.10 | 124 |
|  | *A. maculatus* | 18 | 6 | 25 | 24 |  | 5 | 4 | 44.44 | 9 |
|  | *A. minimus* | 3 |  | 0 | 3 |  |  |  |  |  |
|  | *A. philippinensis* | 7 |  | 0 | 7 |  | 31 | 11 | 26.19 | 42 |
|  | *A. stephensi* | 1 |  | 0 | 1 |  |  |  |  |  |
|  |  |  |  |  |  |  |  |  |  |  |
| **Forest Near Village** | |  |  |  |  |  |  |  |  |  |
|  | *A. aconitus* | 35 | 7 | 16.67 | 42 |  | 43 | 1 | 2.27 | 44 |
|  | *A. barbirostris* | 52 | 9 | 14.75 | 61 |  | 168 | 8 | 4.54 | 176 |
|  | *A. dirus* | 1 | 1 | 50 | 2 |  |  | 5 | 100 | 5 |
|  | *A. hyrcanus* | 23 | 5 | 17.86 | 28 |  | 177 | 13 | 6.84 | 190 |
|  | *A. insulaeflorum* |  |  |  |  |  | 16 | 1 | 5.88 | 17 |
|  | *A. jamesii* | 2 |  | 0 | 2 |  | 10 |  | 0 | 10 |
|  | *A. karwari* |  |  |  |  |  | 9 |  | 0 | 9 |
|  | *A. kochi* | 24 | 5 | 17.241 | 29 |  | 69 | 3 | 4.17 | 72 |
|  | *A. maculatus* | 3 | 2 | 40 | 5 |  | 7 | 3 | 30 | 10 |
|  | *A. minimus* |  |  |  |  |  | 4 |  | 0 | 4 |
|  | *A. philippinensis* | 7 |  | 0 | 7 |  | 161 | 15 | 8.53 | 176 |
|  | *A. tessellatus* |  |  |  |  |  | 1 |  | 0 | 1 |
|  |  |  |  |  |  |  |  |  |  |  |
| **Plantation** |  |  |  |  |  |  |  |  |  |  |
|  | *A. aconitus* |  | 4 |  | 4 |  |  | 1 |  | 1 |
|  | *A. barbirostris* |  | 14 |  | 14 |  |  | 41 |  | 41 |
|  | *A. dirus* |  | 1 |  | 1 |  |  | 6 |  | 6 |
|  | *A. hyrcanus* |  | 14 |  | 14 |  |  | 3 |  | 3 |
|  | *A. maculatus* |  |  |  |  |  |  | 5 |  | 5 |
|  | *A. philippinensis* |  | 5 |  | 5 |  |  | 4 |  | 4 |
|  |  |  |  |  |  |  |  |  |  |  |
| **Village** |  |  |  |  |  |  |  |  |  |  |
|  | *A. aconitus* | 48 | 3 | 5.88 | 51 |  | 53 | 1 | 1.85 | 54 |
|  | *A. barbirostris* | 6 |  | 0 | 6 |  | 47 | 1 | 2.08 | 48 |
|  | *A. dirus* |  |  |  |  |  | 1 | 1 | 50 | 2 |
|  | *A. hyrcanus* | 5 | 2 | 28.57 | 7 |  | 43 | 8 | 15.69 | 51 |
|  | *A. insulaeflorum* |  |  |  |  |  | 1 |  | 0 | 1 |
|  | *A. jamesii* | 3 |  | 0 | 3 |  | 4 | 1 | 20 | 5 |
|  | *A. karwari* |  |  |  |  |  | 2 |  | 0 | 2 |
|  | *A. kochi* | 9 |  | 0 | 9 |  | 45 |  | 0 | 45 |
|  | *A. maculatus* | 6 | 1 | 14.29 | 7 |  | 4 | 3 | 42.86 | 7 |
|  | *A. minimus* |  |  |  |  |  | 7 | 1 | 12.5 | 8 |
|  | *A. philippinensis* | 9 |  | 0 | 9 |  | 311 | 10 | 3.11 | 321 |

| Table S5. Frequency of Insecticide Resistance Allele in Rare Species | | | |  |
| --- | --- | --- | --- | --- |
| **Mosquito Species** | **Sample Size** | ***kdr* L1014F** | ***ace-1* G1119S** | ***rdl* A296S** |
| *A. annularis* | 3 | 0% | 0% | 0% |
| *A. barbirostris* | 2 | 0% | 0% | 50% |
| *A. interruptus* | 1 | 0% | ND* | 0% |
| *A. jamesii* | 16 | 0% | 0% | 44% |
| *A. karwari* | 3 | 0% | 0% | 17% |
| *A. philippinensis* | 17 | 0% | 0% | 15% |
| *A. pseudojamesi* | 1 | 0% | 0% | 0% |
| *A. rampae* | 2 | 0% | 0% | 0% |
| *A. sawadwongporni* | 4 | 0% | 0% | 37.50% |
| *A. sinensis* | 3 | 0% | 100% | 100% |
| *A. splendidus* | 1 | 0% | 0% | 0% |
| *A. tessellatus* | 1 | 0% | 0% | 100% |
| *A. vagus* | 12 | 0% | 0% | 100% |
| Unknown *Anopheles* | 2 | 0% | 0% | 25% |
| ND (not determined) |  |  |  |  |
|  |  |  |  |  |

Table S6. Going to bed time, bet net usage and field activities in the human population.

| *Survey indicators* | |  | n | % | 95%CI |
| --- | --- | --- | --- | --- | --- |
| *Going to bed time* | |  |  |  |  |
|  |  | 17:00pm | 12 | 0.29 | 0.16 |
|  |  | 18:00pm | 284 | 6.89 | 0.77 |
|  |  | 19:00pm | 1362 | 33.04 | 1.44 |
|  |  | 20:00pm | 1292 | 31.44 | 1.42 |
|  |  | 21:00pm | 732 | 17.76 | 1.17 |
|  |  | 22:00pm | 308 | 7.47 | 7.47 |
|  |  | 23:00pm or later | 132 | 3.2 | 0.54 |
|  |  |  |  |  |  |
| *Using a net the night before* | | |  |  |  |
|  |  | Yes | 3880 | 92.38 | 0.8 |
|  | *Time using this bed net* | |  |  |  |
|  |  | Less than 6 months ago | 856 | 22.08 | 1.31 |
|  |  | Last year | 1122 | 28.94 | 1.43 |
|  |  | Last 2 years or more | 1899 | 48.98 | 1.57 |
|  | *Treated bed net* |  | 1594 | 41.24 | 1.55 |
|  |  |  |  |  |  |
| *Using repellent the night before* | | |  |  |  |
|  |  | Yes | 524 | 12.48 | 0.99 |
|  |  |  |  |  |  |
| *Frequency of sleeping under a net* | | |  |  |  |
|  |  | Every night | 3757 | 89.64 | 0.92 |
|  |  | On and off | 395 | 9.42 | 0.88 |
|  |  | Never | 39 | 0.93 | 0.29 |
|  |  |  |  |  |  |
| *Working outside the village* | |  |  |  |  |
|  | Working in the nearby forest | | 147 | 3.5 | 0.56 |
|  |  | Slept overnight | 11 | 7.48 | 4.25 |
|  |  | Used a net | 11 | 100 |  |
|  | Working in the deep forest | | 145 | 3.45 | 0.55 |
|  |  | Slept overnight | 113 | 77.93 | 6.75 |
|  |  | Used a net | 28 | 24.78 | 7.96 |
|  | Working in the rubber plantation | | 296 | 7.05 | 0.77 |
|  |  | Slept overnight | 11 | 3.72 | 2.15 |
|  |  | Used a net | 10 | 90.91 | 16.99 |
|  | Working in the cashew nuts plantation | | 1147 | 27.31 | 1.35 |
|  |  | Slept overnight | 53 | 4.62 | 1.22 |
|  |  | Used a net | 48 | 90.57 | 7.87 |
|  | Working in the cassava field | | 2085 | 49.64 | 1.51 |
|  |  | Slept overnight | 117 | 5.61 | 0.99 |
|  |  | Used a net | 104 | 88.89 | 5.69 |
|  | Working in the rice field | | 328 | 7.81 | 0.81 |
|  |  | Slept overnight | 62 | 18.9 | 4.24 |
|  |  | Used a net | 62 | 100 |  |
